# The combination of an inflammatory peripheral blood gene expression and imaging biomarkers enhance prediction of radiographic progression in knee osteoarthritis

**DOI:** 10.1186/s13075-020-02298-6

**Published:** 2020-09-10

**Authors:** Mukundan Attur, Svetlana Krasnokutsky, Hua Zhou, Jonathan Samuels, Gregory Chang, Jenny Bencardino, Pamela Rosenthal, Leon Rybak, Janet L. Huebner, Virginia B. Kraus, Steven B. Abramson

**Affiliations:** 1 Department of Medicine, NYU Grossman School of Medicine, New York, NY USA; 2 Division of Rheumatology, Rheumatology Research laboratory, NYU Langone Orthopedic Hospital, 301 East 17th Street, Suite 1612, New York, NY 10003 USA; 3 Applied Bioinformatics Laboratories, NYU Grossman School of Medicine, New York, NY USA; 4 Department of Radiology, NYU Grossman School of Medicine, New York, NY USA; 5 Division of Musculoskeletal Imaging, Department of Radiology, Hospital of the University of Pennsylvania, Philadelphia, PA 19104 USA; 6 Duke University School of Medicine, Durham, NC USA

**Keywords:** Osteoarthritis, Radiographic progression, Joint space narrowing, Inflammatory gene expression, Molecular biomarkers, Bone marrow lesion, Magnetic resonance imaging

## Abstract

**Objective:**

Predictive biomarkers of progression in knee osteoarthritis are sought to enable clinical trials of structure-modifying drugs. A peripheral blood leukocyte (PBL) inflammatory gene signature, MRI-based bone marrow lesions (BML) and meniscus extrusion scores, meniscal lesions, and osteophytes on X-ray each have been shown separately to predict radiographic joint space narrowing (JSN) in subjects with symptomatic knee osteoarthritis (SKOA). In these studies, we determined whether the combination of the PBL inflammatory gene expression and these imaging findings at baseline enhanced the prognostic value of either alone.

**Methods:**

PBL inflammatory gene expression (increased mRNA for IL-1β, TNFα, and COX-2), routine radiographs, and 3T knee MRI were assessed in two independent populations with SKOA: an NYU cohort and the Osteoarthritis Initiative (OAI). At baseline and 24 months, subjects underwent standardized fixed-flexion knee radiographs and knee MRI. Medial JSN (mJSN) was determined as the change in medial JSW. Progressors were defined by an mJSN cut-point (≥ 0.5 mm/24 months). Models were evaluated by odds ratios (OR) and area under the receiver operating characteristic curve (AUC).

**Results:**

We validated our prior finding in these two independent (NYU and OAI) cohorts, individually and combined, that an inflammatory PBL inflammatory gene expression predicted radiographic progression of SKOA after adjustment for age, sex, and BMI. Similarly, the presence of baseline BML and meniscal lesions by MRI or semiquantitative osteophyte score on X-ray each predicted radiographic medial JSN at 24 months. The combination of the PBL inflammatory gene expression and medial BML increased the AUC from 0.66 (*p* = 0.004) to 0.75 (*p* < 0.0001) and the odds ratio from 6.31 to 19.10 (*p* < 0.0001) in the combined cohort of 473 subjects. The addition of osteophyte score to BML and PBL inflammatory gene expression further increased the predictive value of any single biomarker. A causal analysis demonstrated that the PBL inflammatory gene expression and BML independently influenced mJSN.

**Conclusion:**

The use of the PBL inflammatory gene expression together with imaging biomarkers as combinatorial predictive biomarkers, markedly enhances the identification of radiographic progressors. The identification of the SKOA population at risk for progression will help in the future design of disease-modifying OA drug trials and personalized medicine strategies.

## Introduction

Osteoarthritis (OA) is a leading cause of pain and morbidity globally, with increasing incidence and prevalence as the population ages [1]. OA is characterized by progressive and often relatively slow deleterious alteration of joint tissues, including cartilage, bone, and synovium [2–7]. Yet, no approved disease-modifying OA drugs (DMOADs) exist that slow progression of the disease. Radiographic progression of knee OA in unselected populations, measured as joint space narrowing (JSN), is low—approximately 0.1–0.15 mm/year [8–10]. However, in such studies, as many as 30–40% of the study population shows no evidence of JSN over 1–2 years, which presents a significant obstacle to DMOAD development [11–15]. To address this challenge, researchers have turned to identify baseline imaging and blood-based prognostic biomarkers that can differentiate progressors from non-progressors among patients with symptomatic knee OA (SKOA).

We have previously shown that increased peripheral blood leukocyte (PBL) gene expression of inflammatory proteins IL-1β, TNFα, and COX-2 (*PBL inflammatory gene expression*) was associated with radiographic progression of knee OA at 24 months [15–19]. Similarly, separate studies indicate that the presence of bone marrow lesions (BML) on MRI is an imaging biomarker that identifies patients at higher risk for progression [20–22]. Although each of these biomarkers has demonstrated individual utility, no studies have focused on whether a combination of inflammatory and imaging biomarkers improves prediction of radiographic progression more than a single biomarker alone [23–27]. Maximizing the predictive value of baseline biomarkers will enable the powering and reduce the cost of future DMOAD studies [28].

The analyses reported here represent an extension of our existing cohort to 243 patients from a previous study of 111 patients, as well as an analysis of 204 SKOA patients selected from the OA Initiative (OAI). We determined the prognostic utility of the baseline PBL inflammatory gene expression, and MRI images, alone and in combination, as predictive biomarkers of SKOA radiographic progression. Additionally, we employed predictive multivariable models of a dichotomized outcome and used the area under the receiver operating characteristic curves (AUCs) to assess the predictive performance of baseline biomarkers to determine those most predictive of 24-month radiographic JSN. Our main aim was to identify baseline combinatorial biomarker(s) to predict and identification of patients at risk for “fast progression” of radiographic SKOA.

## Patients and methods

### Patient population

#### NYU cohort

**Fig. 1:**
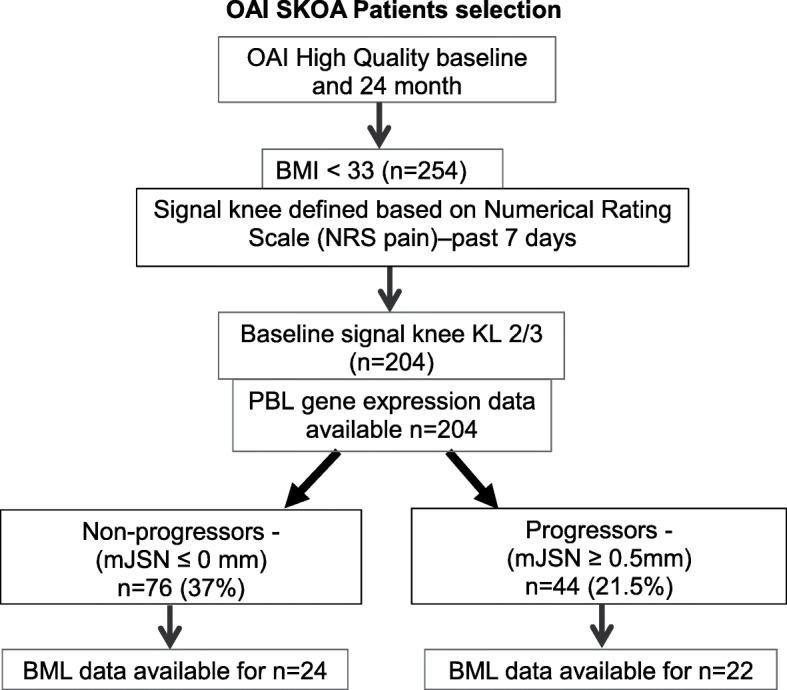
Osteoarthritis Initiative symptomatic knee OA selection criteria for PBL gene expression and bone marrow lesion studies. PBL inflammatory gene expression data were available for all (*n* = 204) patients and MRI–BML data was available only for *n* = 46 patients

Based on a priori knowledge, we expect a minimum of 30% of participants to progress (defined as JSN ≥ 0.5 mm/24 months) and a minimum of 30% to show no evidence of progression (JSN = 0 mm/24 months. Therefore, we recruited 132 additional patients to our prior cohort of 111 patients with SKOA (*n* = 243) who completed a 24-month NIH-funded prospective study evaluating biomarkers in OA [11, 12] (Fig. 1), satisfying a power analysis to detect an effect size of 0.3 for biomarkers at significance level of 0.05 and power of 0.85.

### NYU cohort inclusion and exclusion criteria

As part of an NIH-funded study, we recruited and followed for 24 months SKOA patients who met American College of Rheumatology (ACR) knee OA radiographic criteria [Kellgren–Lawrence (KL) grade ≥ 1] and clinical symptomatic criteria with at least 3 of the following: age > 50 years, stiffness < 30 min, crepitus, bony tenderness, bony enlargement, no palpable warmth [25]. Patients having any of the following were excluded: any other form of arthritis (including rheumatoid arthritis, spondyloarthritis, active crystal arthropathy); body mass index (BMI) ≥ 33; any disorder requiring the use of systemic corticosteroids within 1 week of screening, history of bilateral knee replacements; major co-morbidities including diabetes mellitus, non-cutaneous cancer within 5 years of screening, chronic hepatic or renal disease, chronic infectious disease, congestive heart failure; and hyaluronan and/or corticosteroid injections to the affected knee within 3 months of screening. Some exclusion criteria, such as the BMI cutoff, were chosen to mitigate potential effects of the covariate on inflammatory peripheral blood leukocyte (PBL) gene expression markers, which were investigated as a separate aim of this study and reported upon elsewhere [17]. The Institutional Review Board at NYU Medical Center approved the protocol. Informed consent was obtained from all subjects.

### Clinical assessments

All subjects had completed the visual analog scale (VAS) and WOMAC pain assessments at baseline and every 6 months for the duration of the study. Pain questions were specific to the more painful knee (signal knee). Subjects also had a physical exam by a study physician at baseline and after 24 months.

### NYU imaging: radiograph and MRI acquisition and scoring

#### Knee radiographs

Subjects underwent standardized weight-bearing fixed-flexion posteroanterior knee radiographs using the SynaFlexer™ X-ray positioning frame (Synarc) at baseline and 24 months as described previously [16, 29]. The radiographic beam angle was optimized for the medial joint space compartment. Radiographic readings were done separately by two musculoskeletal radiologists (LR, JB) blinded to patient demographics, clinical information, and MRI readings. X-rays were scored for KL grade 0–4 [30], and medial and lateral joint space width (JSW) measured at the mid-portion of the joint space via electronic calipers. Joint space narrowing (JSN) was calculated as the change in JSW, in millimeters, from baseline to 24-month follow-up. Disagreements between the two readers were resolved by consensus. Cohen’s kappa coefficient for interrater agreement on KL grades was 0.85 and 0.77 for the right knee and left knee, respectively, and kappas for JSW were ≥ 0.93 for medial compartments of both the right and left knees. Based on the high inter-reader correlations, a single reader (LR) was employed for the 24-month follow-up. Osteophyte scoring—both medial and lateral osteophytes in tibial and femoral regions scored semiquantitatively (0–3) [0 = absent, 1 = mild, 2 = moderate, and 3 = severe]. Since the majority of both the NYU and OAI subjects (approximately 80%) had medial compartment disease, we restricted our analysis to medial radiographic JSN progressors.

#### NYU knee MR imaging protocol

Of the 243 subjects enrolled, only 111 subjects had MR imaging performed [on a 3.0T clinical scanner (Magnetom Tim Trio; Siemens Medical Solutions, Erlangen, Germany) using an eight-channel transmit-receive phased-array knee coil (In vivo Corporation, FL)]. The knee imaging protocol consisted of a sagittal 3D-high resolution T1-weighted-fast low angle shot (FLASH) sequence with selective water excitation (TR/TE = 25/4 ms; flip angle = 25; FOV = 15 × 15 cm; slice thickness = 1.5 mm; matrix = 512 × 384; receiver bandwidth = 200 Hz/pixel) as well as sagittal T2-weighted fat-saturated spin echo (TR/TE = 4000/75 ms; FOV = 15 × 15 cm; slice thickness = 3 mm; matrix = 256 × 128; receiver bandwidth = 130 Hz/pixel).

#### NYU knee MR assessments: WORMS scoring

A musculoskeletal radiologist (GC), blinded to the clinical and radiographic information, but not blinded to acquisition time point, performed Whole-Organ Magnetic Resonance Imaging Score (WORMS) scoring on sagittal T2-weighted fat-saturated images and sagittal T1-weighted 3-D spoiled gradient-echo images [31]. Specifically, cartilage morphology (score of 0–6) and subarticular bone marrow lesions (BML; a score of 0–3) were scored within the anterior, central, and posterior regions of the medial and lateral tibial plateaus; central and posterior regions of the medial and lateral femoral condyles were also scored. Medial and lateral meniscal morphology (score of 0–4) were also evaluated. Interrater reliability was assessed by scoring 10 subjects in two separate sessions, 1 week apart. Paired *t*-tests, applied for assessing differences for cartilage, BML, and meniscus readings (*p* values 0.354, 0.797, and 0.766 for cartilage, BML and meniscus readings, respectively), showed that there were no significant differences, which verified reading reliability of our data. Cartilage medial scores were calculated as the sum of the medial femur central, medial femur posterior, medial tibia anterior, medial tibia central, and medial tibia posterior regions. Cartilage lateral scores were calculated as the sum of lateral femur central, lateral femur posterior, lateral tibia anterior, lateral tibia central, and lateral tibia posterior regions. Overall, cartilage scores were calculated by summing lateral and medial cartilage scores. BML medial scores were calculated as the sum of the medial femur central, medial femur posterior, medial tibia anterior, medial tibia central, and medial tibia posterior regions. BML lateral scores were calculated as the sum of lateral femur central, lateral femur posterior, lateral tibia anterior, lateral tibia central, and lateral tibia posterior regions. Overall, BML scores were calculated by summing lateral and medial BML scores.

#### The OAI cohort

The OAI is a multi-center, longitudinal, prospective observational study of knee OA. The main goal of the OAI is to develop a public domain research resource to facilitate the scientific evaluation of biomarkers for knee OA as potential surrogate endpoints for disease onset and progression. Participants were selected from the Osteoarthritis Initiative (OAI; http://www.oai.ucsf.edu/), a longitudinal cohort of 4796 participants with clinical, radiological, biochemical, and other data collected at baseline and annual follow-up visits. OAI recruited participants with SKOA, and also those with no OA but considered at high risk of incident OA. Clinical, radiographic, and MRI data were obtained from the OAI database (https://data-archive.nimh.nih.gov/oai). MRI images were scored for BMLs using the semiquantitative (SQ) (MRI Osteoarthritis Knee Score) MOAKS system available at the OAI site. For each subregion, MOAKS scores three features using an ordinal score for size, number of BMLs, and percentage of lesion that is a BML. The OAI dataset includes both MRI and radiographic images. Baseline clinical data, MRI BML scores, radiographs (baseline and 24 months), and buffy coat samples for PBL gene expression studies were obtained.

##### OAI cohort: high-quality OAI (HQ-OAI) radiographs

The OAI imaging acquisition techniques and reading protocols are publicly available at http://oai.epi-ucsf.org/datarelease. Baseline OA severity was assessed on knee radiographs centrally read and graded according to the Kellgren–Lawrence (KL) system [30]. Briefly, bilateral posteroanterior fixed-flexion weight-bearing radiographic views were obtained using a SynaFlexerTM frame (Synarc, Newark, CA, USA). The detailed Radiographic Procedure Manual is available online (https://oai.epiucsf.org/datarelease/operationsManuals/ RadiographicManual.pdf). We selected a cohort of 443 cases, whose knee radiographs had high-quality MTP alignment (defined as the inter-margin distance (IMD) of ≤ 1.70 mm at baseline and 24-month films [32, 33]. Furthermore, from this high-quality sub-cohort, we have selected patients whose BMI is < 33 and signal knee (painful knee) with KL 2 or 3 (*n* = 204) were selected for this study (Fig. 1).

##### MR image acquisition and quantitative measures

Non-contrast MRIs were obtained on 3T Trio systems (Siemens Healthcare, Erlangen, Germany), and the complete pulse sequence protocol and sequence parameters have been described previously [34].

##### BML MOAKS Score

MRI BML scores available for all the subjects for whom PBL inflammatory gene expression data available were downloaded from the OAI site. Briefly, BML was scored using the semiquantitative MRI Osteoarthritis Knee Score (MOAKS) system, which is available at the OAI site [35]. For each subregion, MOAKS scores three features using an ordinal score for size, number of BMLs, and percentage of lesion that is a BML. The utilization of study protocol and biospecimens were reviewed and approved by the NYU School of Medicine IRB.

##### Radiographic progression

For the medial JSN outcome variable, our definition of radiographic progression was similar to the case definitions described previously based on previously published reports [8, 10, 27]. SKOA patients who had narrowing in the medial tibiofemoral compartment by at least 0.5 mm over 24 months from baseline in the signal knee. We defined non-progressors or no progression as no increase, defined as JSN ≤ 0.0 mm over 24 months.

### Sample collection and assessment

#### PBL isolation and inflammatory gene expression

##### NYU cohort

At the time of baseline knee radiographs, non-fasting blood samples were collected in pyrogen-free heparinized tubes for PBL isolation using the Ficoll-Hypaque density gradient centrifugation. Total RNA was isolated from PBLs and from citrate buffy coats (OAI cohort) using the Qiagen RNeasy Kit (Qiagen) as described previously [15, 17]. For both NYU and OAI studies, relative expression of inflammatory mRNA expression in PBLs was determined using Predesigned TaqMan primer sets (IL1B – Hs00174097_m1; TNFA – Hs00174128 _m1; PTGS2 (COX-2) – Hs00153133_m1) (Applied Biosystems). qPCR was performed as previously described [15], normalized against housekeeping genes GAPDH and 18S, and fold-change was calculated using the delta Ct method [36]. For the OAI study, the relative fold-change data were calculated against super-control (*n* = 100) obtained from OAI biorepository who did not develop knee OA over 8 years of follow-up and the qPCR obtained CT values of each target(s) including the housekeeping genes were shared with the OAI biorepository team for de-identification and the following association studies.

### Statistical methods

The relationships between baseline clinical, demographic, and imaging variables, including Western Ontario and McMaster Universities Osteoarthritis Index (WOMAC) pain, visual analog scale (VAS) pain, radiographic JSW, osteophytes, MRI-determined cartilage and BML scores, age, sex, and BMI were determined by Spearman’s correlation. Associations between variables were assessed by partial correlations controlling for age, sex, and BMI. Support vector machines were used for building predictive multivariate models of a dichotomized outcome variable based on multiple biomarkers [37]. The null model against which all other predictive models are compared and *p* values are calculated is the random model (i.e., coin-flipping model), and the random model has AUC of 0.5. For estimating future predictivity of multivariate predictive models, we used 10-fold stratified cross-validation repeated with 100 different splits of data into 10-folds [38]. We used area under receiver operating characteristic curve (AUC) and logistic regression (either unadjusted or adjusted for age, sex, BMI) model to investigate the prognostic value of PBL gene expression, radiographic osteophytes, MRI-BML, and cartilage score for predicting JSN.

This multivariate method can fit both simple and complex functions, avoids overfitting by using effective regularization strategies, and has shown excellent empirical performance in a wide range of biomedical predictive modeling applications [39]. AUC values were compared against random models for significance using Delong’s test [40]. False discovery rate (FDR) was used to adjust the *p* values for multivariate comparison [41]. To evaluate whether medial BML as an additional predictor improved the regression model, we used 2 methods: (1) Delong’s test comparing the AUCs from the model with biomarkers plus medial BML against the model with biomarkers alone, and (2) ANOVA comparing linear regression models of JSN with biomarkers plus medial BML versus biomarkers alone.

Causal graph analysis was performed, for which we used the FCI algorithm in the TETRAD software package (http://www.phil.cmu.edu/projects/tetrad/version 4.3.10-7). This method is capable of discovering a causal graph that most closely resembles the data distribution [9]. Independence testing was based on Fisher’s *Z* test, with the significance level set to 0.10. No data manipulation of any kind (e.g., transformation, imputation, thresholding) was applied; therefore, these analyses were not biased toward particular causal hypotheses.

## Results

### An inflammatory *PBL gene expression* predicts fast radiographic progressors in both the NYU and OAI cohort

We recruited a total of 243 SKOA patients, followed in the clinics of NYU and analyzed the PBL inflammatory gene expression as a predictive biomarker of radiographic progression. The baseline demographic and clinical characteristics of these subjects are summarized in Table 1. Of the 243 patients in the NYU cohort, 30% exhibited ≥ 0.5 mm mJSN at 24 months in the signal knee and were designated “fast progressors” [8, 10, 42]. PBL mRNA expression of IL-1β, COX-2, and TNFα, at baseline significantly predicted fast radiographic progressors with AUCs that ranged from 0.62 to 0.68 (*p* = 0.003 to < 0.0001) (Table 2).

**Table 1:** NYU and OAI cohorts SKOA subject baseline demographics and imaging features

Variable	NYU (***n*** = 243)	OAI (***n*** = 203)
Age (years)	60.1 (10.6)	62.8 (10.6)
Sex (%):		
Male	33.30%	47.0%
Female	66.70%	53.0%
BMI (kg/m^2^)	26.7 (3.6)	27.3 (3.1)
VAS (0–100)	42.3 (29.9)	NA
WOMAC (0–100)	36.6 (24.7)	18.5 (16.2)
**Radiographic joint space width (JSW) (mm):**		
Baseline	3.65 (1.34)	3.62 (1.30)
24 months	3.13 (1.51)	3.36 (1.49)
**Radiographic osteophytes:**		
Medial tibial plateau (MTP) (0–3)	0.50 (0.70)	1.08 (1.17)
Medial femoral condyle (MFC) (0–3)	0.87 (0.69)	0.93 (1.02)
Lateral tibial plateau (LTP) (0–3)	0.41 (0.64)	0.99 (0.80)
Medial femoral condyle (LFC) (0–3)	0.51 (0.60)	0.33 (0.38)
**MRI:**	**(*n*** **= 111)**	**(*n*** **= 46)**
Mean medial BML WORMS score (0–15)	1.13 (1.86)	1.90 (2.04)

Data shown are the mean (SD), total number or percent affected, as indicated. *SKOA* symptomatic knee OA, *BMI* body mass index, *VAS* visual analog scale, *WOMAC* Western Ontario and McMaster Universities Osteoarthritis Index, *WORMS* Whole-Organ Magnetic Resonance Imaging Score, *BML* bone marrow lesion, *NA* not available. Both medial and lateral osteophytes in tibial plateau (MTP, LTP) and femoral condyle (MFC, LFC) regions scored semiquantitatively (0–3) [0 = absent, 1-mild, 2-moderate, and 3 = severe]. Subarticular bone marrow lesions (BML, a score of 0–3) were scored within the anterior, central, and posterior regions of the medial and lateral tibial plateaus; central and posterior regions of the medial and lateral femoral condyles were also scored

**Table 2:** The area under the receiver operating characteristic curves (AUC) of inflammatory PBL gene expression biomarkers to radiographic fast progressors of JSN (> 0.5 mm) at 24 months in patients with SKOA in NYU extended and OAI cohorts

Fast progressors (JSN > 0.5 mm) vs. non-progressors (JSN ≤ 0.0 mm)	AUC (95% CI)	Adjusted ***p*** value for model
**NYU (*n*** **= 243)**		
Age	0.48 (0.39–0.56)	0.711
Sex	0.62 (0.54–0.71)	0.001
BMI	0.61 (0.53–0.69)	0.004
Age + sex + BMI (ASB)	0.65 (0.57–0.73)	< 0.0001
IL-1β	0.62 (0.53–0.70)	0.003
COX-2	0.68 (0.60–0.75)	< 0.0001
TNFα	0.66 (0.57–0.74)	< 0.0001
IL-1β + COX-2 + TNFα	0.60 (0.5 2–0.69)	0.008
IL-1β + ASB	0.65 (0.57–0.73)	< 0.0001
COX-2 + ASB	0.66 (0.58–0.74)	< 0.0001
TNFα + ASB	0.64 (0.56–0.72)	< 0.0001
IL-1β + COX-2 + TNFα + ASB	0.66 (0.58–0.74)	< 0.0001
**OAI (*n*** **= 203)**		
Age	0.38 (0.27–0.49)	0.987
Sex	0.50 (0.39–0.61)	0.482
BMI	0.38 (0.27–0.48)	0.989
Age + sex + BMI (ASB)	0.36 (0.26–0.47)	0.995
IL-1β	0.76 (0.66–0.85)	< 0.0001
COX-2	0.64 (0.53–0.75)	0.006
TNFα	0.68 (0.58–0.79)	< 0.0001
IL-1β + COX-2 + TNFα	0.69 (0.59–0.79)	< 0.0001
IL-1β + ASB	0.63 (0.53–0.73)	0.007
COX-2 + ASB	0.50 (0.38–0.61)	0.513
TNFα + ASB	0.56 (0.45–0.67)	0.158
IL-1β + COX-2 + TNFα + ASB	0.59 (0.48–0.69)	0.063
**NYU + OAI combined (*n*** **= 436)**		
Age	0.47 (0.40–0.54)	0.822
Sex	0.56 (0.49–0.63)	0.039
BMI	0.57 (0.50–0.64)	0.021
Age + sex + BMI (ASB)	0.57 (0.50–0.63)	0.019
IL-1β	0.66 (0.60–0.72)	< 0.0001
COX-2	0.66 (0.59–0.72)	< 0.0001
TNFα	0.67 (0.60–0.73)	< 0.0001
IL-1β + COX-2 + TNFα	0.63 (0.57–0.70)	< 0.0001
IL-1β + ASB	0.60 (0.54–0.67)	0.001
COX-2 + ASB	0.59 (0.53–0.66)	0.003
TNFα + ASB	0.57 (0.50–0.64)	0.019
IL-1β + COX-2 + TNFα + ASB	0.61 (0.54–0.68)	0.001

*ASB* age, sex, and BMI, *JSN* joint space narrowing, *SKOA* symptomatic knee OA, *AUC* area under the (receiver operating characteristic) curve, *95% CI* 95% confidence interval; All comparisons are versus non-progressors (JSN ≤ 0 mm). NYU cohort: non-progressors (*n* = 115) and fast progressors (*n* = 72); OAI cohort: non-progressors (*n* = 76) and fast progressors (*n* = 44)

To replicate these NYU Cohort PBL transcriptomic studies in an independent population, we next examined radiographic progression in an OAI Cohort (*n* = 204) of subjects with symptomatic knee osteoarthritis. “Fast progression” was observed in 22% of the OAI Cohort. In the NYU cohort, age as covariant did not have significant predictive power, whereas sex and BMI did have significant but moderate AUC values 0.62 and 0.61 (*p* = 0.001 and 0.004) (Table 2) in predicting fast progressors. In contrast, in the OAI cohort, these variants did not achieve significance. We note that while age and BMI were similar in both cohorts, the OAI population had a higher percentage of females (NYU 33%; OA 47%). As shown in the combined cohort, both sex and BMI retained significance but with moderate predictive power in predicting fast progressors. Increased mRNA expression of IL-1β, COX-2, and TNFα, (levels between non-progressors and progressors are presented in supplemental Table 1) significantly predicted JSN fast progressors (≥ 0.5 mm at 2 years) with AUCs that ranged from 0.64 to 0.76 (*p* = 0.006 to < 0.0001) (Table 2). In the combined NYU and OAI cohort of 447 subjects, the significance of an inflammatory PBL gene expression was retained (Table 2) in predicting fast radiographic progressors (AUC 0.66 to 0.67 (*p* < 0.0001)).

### MRI cross-sectional imaging, radiographic, and symptom relationships

The association of baseline MRI-scored variables with clinical and radiographic features at baseline and 2 years is shown in Table 3. Baseline medial tibial central BML scores associated significantly, but modestly, with baseline WOMAC scores for pain (*r* = 0.19, *p* = 0.048) and 24-month VAS pain reports (*r* = 0.20, *p* = 0.043) (Table 3). Additionally, medial tibial central BML associated moderately with baseline KL scores and associated inversely with baseline JSW (Table 3, *r* = 0.21, *p* < 0.01; *r* = − 0.22, *p* = 0.018, respectively). Cartilage scores in all WORMS medial subregions, separately and/or summed, were also associated with baseline KL scores (*r* values ranging from 0.27 to 0.34 depending on the specific subregion, all *p* values < 0.01; Table 3). Similarly, cartilage scores in nearly all medial subregions were inversely associated with baseline JSW (*r* values ranging from − 0.27 to − 0.33, all *p* values < 0.01). Meniscus overall readings were also associated with baseline WOMAC pain and associated inversely with mJSW at 24 months (*r* = − 0.34, *p* < 0.01).

**Table 3:** Association of baseline MRI-scored medial WORMS variables with clinical and radiographic features at baseline and 24 months—NYU cohort (*n* = 111)

	Baseline WOMAC pain	Baseline VAS pain	24-month WOMAC pain	24-month VAS pain	Baseline signal KL	24-month signal KL	Delta signal KL	Baseline medial JSW	24-month medial JSW	Medial JSN
Cartilage overall	0.09 (0.34)	0.01 (0.94)	0.07 (0.45)	0.10 (0.29)	**0.33 (< 0.01)**	**0.44 (< 0.01)**	**0.20(0.03)**	**− 0.28 (< 0.01)**	**− 0.34 (< 0.01)**	0.13 (0.15)
Cartilage medial overall	0.15 (0.12)	0.12 (0.22)	0.09 (0.34)	0.09 (0.38)	**0.36 (< 0.01)**	**0.44 (< 0.01)**	0.14 (0.15)	**− 0.32 (< 0.01)**	**− 0.41 (< 0.01)**	0.10 (0.29)
BML overall	0.07 (0.45)	0.09 (0.36)	0.04 (0.66)	0.10 (0.28)	**0.31 (< 0.01)**	**0.35 (< 0.01)**	0.05 (0.58)	−0.13 (0.16)	**−0.22 (0.02)**	0.14 (0.14)
BML medial overall	0.10 (0.30)	0.13 (0.18)	0.04 (0.69)	0.07 (0.48)	**0.31 (< 0.01)**	**0.32 (< 0.01)**	−0.003 (0.98)	−0.17 (0.07)	**− 0.28 (< 0.01)**	**0.19 (0.05)**
Meniscus overall	**0.20 (0.04)**	0.15 (0.11)	0.08 (0.38)	0.13 (0.18)	**0.30 (< 0.01)**	**0.41 (< 0.01)**	0.16 (0.10)	**−0.24 (0.01)**	**−0.34 (< 0.01)**	**0.23 (0.02)**
Cartilage medial femur central	0.13 (0.17)	0.15 (0.12)	0.02 (0.87)	0.01 (0.90)	**0.27 (< 0.01)**	**0.36 (< 0.01)**	0.16 (0.09)	**−0.31 (< 0.01)**	**− 0.40 (< 0.01)**	0.08 (0.42)
Cartilage medial femur posterior	0.12 (0.22)	0.08 (0.44)	0.15 (0.13)	0.12 (0.21)	**0.34 (< 0.01)**	**0.42 (< 0.01)**	0.13 (0.19)	**−0.33 (< 0.01)**	**− 0.35 (< 0.01)**	0.04 (0.67)
Cartilage medial tibial anterior	0.15 (0.13)	0.11 (0.28)	0.09 (0.42)	0.08 (0.44)	**0.32 (< 0.01)**	**0.39 (< 0.01)**	0.13 (0.16)	**−0.27 (< 0.01)**	**− 0.32 (< 0.01)**	0.03 (0.78)
Cartilage medial tibial central	0.13 (0.17)	0.17 (0.08)	0.11 (0.25)	0.12 (0.20)	**0.29 (< 0.01)**	**0.37 (< 0.01)**	0.15 (0.11)	**−0.31 (< 0.01)**	**− 0.41 (< 0.01)**	0.12 (0.21)
Cartilage medial tibial posterior	−0.04 (0.68)	− 0.07 (0.50)	− 0.09 (0.38)	−0.06 (0.53)	**0.27 (< 0.01)**	**0.31 (< 0.01)**	0.07 (0.48)	−0.08 (0.38)	**− 0.19 (0.05)**	0.05 (0.61)
BML medial femur central	0.03 (0.77)	0.09 (0.34)	−0.05 (0.63)	0.03 (0.74)	0.17 (0.08)	**0.22 (0.02)**	0.07 (0.45)	−0.10 (0.28)	**−0.21 (0.03)**	0.10 (0.30)
BML medial femur posterior	0.06 (0.53)	0.004 (0.96)	0.03 (0.79)	0.10 (0.28)	0.09 (0.35)	0.12 (0.22)	0.03 (0.75)	−0.05 (0.62)	−0.10 (0.29)	0.11 (0.24)
BML medial tibial anterior	**0.19 (0.05)**	0.18 (0.07)	0.09 (0.38)	0.11 (0.28)	0.04 (0.65)	0.08 (0.43)	0.05 (0.64)	−0.05 (0.61)	**−0.19 (0.05)**	**0.22 (0.02)**
BML medial tibial central	**0.19 (0.05)**	0.15 (0.12)	0.15 (0.13)	**0.20 (0.04)**	**0.21 (0.03)**	**0.28 (< 0.01)**	0.09 (0.35)	**−0.22 (0.02)**	**−0.34 (< 0.01)**	**0.22 (0.02)**
BML medial tibial posterior	−0.04 (0.72)	0.02 (0.80)	−0.05 (0.61)	−0.06 (0.56)	0.11 (0.24)	0.13 (0.16)	0.04 (0.70)	−0.03 (0.73)	0.00 (0.97)	−0.01 (0.94)

Data shown are Spearman correlation coefficients, adjusted for age, sex, and BMI. *p* values for the correlations are in parentheses. Significant *p* values are presented in bold font. *WORMS* Whole-Organ Magnetic Resonance Imaging Score, *WOMAC* Western Ontario and McMaster Universities Osteoarthritis Index, *VAS* visual analog scale, *KL* Kellgren–Lawrence grade, *JSW* joint space width, *JSN* joint space narrowing, *BML* bone marrow lesion

### Baseline MRI medial BML (mBML) and meniscus scores predict the progression of radiographic findings

BML detected by MRI has been associated with radiographic progression of knee OA [20–22, 43]. We analyzed MRI findings in our two OA cohorts to determine whether (1) MRI findings predicted the subset of fast progressors (JSN > 0.5 mm). We note that in the NYU cohort, MRI was available for the initial 111 enrollees previously described [12]. Consistent with prior literature, fast progressors (≥ 0.5 mm) in the NYU cohort had significantly higher baseline medial BML scores (1.78 ± 2.22) than non-progressors (0.59 ± 1.14; *p* < 0.014; Table 4A). Similarly, in the OAI cohort, medial BML scores were also higher in the fast progressors cohort (1.21 vs. 3.27; *p* < 0.013).

**Table 4:** Baseline cartilage and BML scores in radiographic non-progressors compared to fast progressors in NYU (4A) and OAI (4B) SKOA

**A—NYU**	**Non-progressors JSN ≤ 0 mm (*n*** **= 39)** **Mean (SD)**	**Fast progressors JSN ≥ 0.5 mm (*n*** **= 45)** **Mean (SD)**	***p*** **value**	**FDR**
Cartilage medial overall (0–30)	13.88 (8.61)	15.42 (8.71)	0.42	0.42
Cartilage lateral overall (0–30)	3.56 (6.62)	5.24 (7.89)	0.298	0.398
BML medial overall (0–15)	0.59 (1.14)	1.78 (2.22)	**0.003**	**0.014**
BML lateral overall (0–15)	0.33 (0.80)	0.16 (0.47)	0.214	0.398
**B—OAI**	**Non-progressors JSN ≤ 0 mm (*n*** **= 29)** **Mean (SD)**	**Fast progressors JSN ≥ 0.5 mm (*n*** **= 34)** **Mean (SD)**	***p*** **value**	**FDR**
Cartilage loss medial (0–36)	4.21 (2.90)	7.09 (3.78)	**0.006**	**0.014**
Cartilage loss lateral (0–36)	5.04 (4.09)	2.32 (2.80)	0.012	0.025
BML medial overall (0–36)	1.21 (1.82)	3.27 (2.31)	**0.002**	**0.013**
BML lateral overall (0–36)	2.23 (2.84)	0.98 (1.50)	0.072	0.102

Abbreviations: *WORMS* Whole-Organ Magnetic Resonance Imaging Score, *BML* bone marrow lesion, *JSN* joint space narrowing, *FDR* false discovery rate. JSN values are expressed in mm as mean ± standard deviation (SD). All comparisons are versus non-progressors (JSN ≤ 0 mm). Significant *p* values are represented in bold font

In the OAI cohort, “Cartilage Loss Medial” was also associated with progression, though this was not observed in the NYU cohort (Table 4B). In the combined cohort, association of medial BML alone with mJSN is also shown in Table 5 (AUC = 0.59; 95% CI (0.51–0.67; *p* < 0.035). The odds ratio (OR) for fast progression associated with medial BML was 2.43 (95% CI 1.44–4.08; *p* < 0.0001) (Table 6). In the NYU cohort, the MRI meniscus score correlated (*r* = 0.23; *p* = 0.02) with mJSN (Table 3), though AUC (0.56; *p* = 0.178) was modest and not significant in the dichotomized radiographic progression analysis (progressors vs. non-progressors). When meniscal findings were combined with BML and PBL inflammatory gene expression, the AUC for either alone increased significantly to 0.73 (*p* < 0.0001; Table 7).

**Table 5:** The area under the receiver operating characteristic curves (AUC) comparing baseline PBL inflammatory gene expression biomarkers, with and without medial BML, to predict 24-month fast radiographic knee OA progression in the combined NYU and OAI cohorts

NYU + OAI combined–biomarkers			Biomarkers + baseline medial BML			
Fast progressors (JSN > 0.5 mm) vs. non-progressors (JSN ≤ 0.0 mm)	AUC (95% CI)	Adjusted ***p*** value for model	Fast progressors (JSN > 0.5 mm) vs. non-progressors (JSN ≤ 0.0 mm)	AUC (95% CI)	***p*** value for model	***p*** value for model performance increase
Age	0.46 (0.38–0.54)	0.27	Age	0.62 (0.54–0.69)	0.005	0.002
Sex	0.52 (0.44–0.60)	0.31	Sex	0.60 (0.52–0.68)	0.006	0.002
BMI	0.54 (0.46–0.62)	0.27	BMI	0.61 (0.53–0.69)	0.005	0.041
ASB	0.50 (0.42–0.58)	0.498	ASB	0.59 (0.51–0.67)	0.014	0.016
COX-2	0.61 (0.53–0.68)	0.015	COX-2	0.65 (0.58–0.73)	< 0.0001	0.074
IL-1β	0.57 (0.49–0.65)	0.081	IL-1β	0.62 (0.54–0.70)	0.004	0.088
TNFα	0.50 (0.42–0.58)	0.498	TNFα	0.59 (0.51–0.67)	0.020	0.021
IL1β + COX2	0.56 (0.48–0.64)	0.094	IL1β + COX2	0.61 (0.54–0.69)	0.005	0.085
IL1β + TNFα	0.52 (0.43–0.60)	0.381	IL1β + TNFα	0.60 (0.52–0.68)	0.011	0.024
COX2 + TNFα	0.57 (0.49–0.65)	0.081	COX2 + TNFα	0.62 (0.54–0.69)	0.005	0.136
IL-1β + COX-2 + TNFα	0.62 (0.54–0.69)	0.007	IL-1β + COX-2 + TNFα	0.66 (0.58–0.73)	< 0.0001	0.114
Baseline BML	0.59 (0.51–0.67)	0.035	Baseline BML			
Osteophytes MFC	0.47 (0.39–0.55)	0.309	Osteophytes MFC	0.57 (0.49–0.65)	0.035	0.010
Osteophytes MTP	0.48 (0.40–0.56)	0.356	Osteophytes MTP	0.58 (0.50–0.66)	0.035	0.011
Osteophytes LFC	0.58 (0.50–0.66)	0.072	Osteophytes LFC	0.61 (0.53–0.69)	0.005	0.096
Osteophytes LTP	0.47 (0.39–0.55)	0.309	Osteophytes LTP	0.58 (0.50–0.66)	0.029	0.007
Osteophytes MFC + MTP	0.46 (0.38–0.54)	0.207	Osteophytes MFC + MTP	0.57 (0.48–0.65)	0.056	0.006
Osteophytes LFC + LTP	0.57 (0.49–0.65)	0.081	Osteophytes LFC + LTP	0.61 (0.53–0.69)	0.006	0.083
ALL osteophytes	0.57 (0.49–0.65)	0.081	ALL osteophytes	0.61 (0.53–0.69)	0.005	0.064
IL-1β + COX-2 + TNFα + osteophytes MFC + MTP	0.59 (0.51–0.67)	0.038	IL-1β + COX-2 + TNFα + osteophytes MFC + MTP	0.63 (0.55–0.71)	0.002	0.114
IL-1β + COX-2 + TNFα + osteophytes LFC + LTP	0.67 (0.59–0.74)	< 0.0001	IL-1β + COX-2 + TNFα + osteophytes LFC + LTP	0.68 (0.60–0.76)	< 0.0001	0.271
IL-1β + COX-2 + TNFα + ALL osteophytes	0.67 (0.59–0.74)	< 0.0001	IL-1β + COX-2 + TNFα + ALL osteophytes	0.68 (0.61–0.76)	< 0.0001	0.223
ALL markers	0.68 (0.61–0.76)	< 0.0001				

*ASB* age, sex, and BMI, *PBL* peripheral blood leukocyte, *BML* bone marrow lesion, *JSN* joint space narrowing, *COX-2* cyclooxygenase-2, *IL-1β* interleukin-1 beta, *TNFα* tumor necrosis factor alpha, *95% CI* 95% confidence intervals. Medial (MTP) and lateral (LTP) osteophytes in tibial plateau and medial (MFC) and lateral (LFC) femoral condyle. All comparisons are versus non-progressors (JSN ≤ 0 mm). Significant *p* values are represented in bold font. Total number of progressors (*n* = 85) and non-progressors (*n* = 115) in the combined cohort

**Table 6:** The odds ratios (OR) comparing baseline inflammatory *PBL inflammatory gene expression* biomarkers and osteophytes with and without medial BML to predict 24-month fast radiographic knee OA progression in the combined NYU and OAI cohorts

Biomarkers				Biomarkers + baseline medial BML				
Fast progressors (JSN > 0.5 mm) vs. non-progressors (JSN ≤ 0.0 mm)	Odds ratio	95% CI	Adjusted ***p*** value for model	Biomarkers + baseline medial BML	Odds ratio	95% CI	Adjusted ***p*** value for model	***p*** value for increase
Age	1	(1.0–1.0)	0.769	Age	2.46	(1.32–4.6)	0.006	0.001
Sex	1	(1.0–1.0)	0.678	Sex	2.29	(1.24–4.23)	0.007	0.002
BMI	1	(1.0–1.0)	0.151	BMI	2.15	(1.17–3.96)	0.006	0.005
COX-2	3.56	(0.92–13.72)	0.019	COX-2	8.64	(1.36–54.84)	< 0.0001	< 0.0001
IL-1β	1.90	(0.56–6.41)	0.074	IL-1β	4.09	(0.753–22.230)	< 0.0001	< 0.0001
TNFα	1.12	(0.78–1.63)	0.696	TNFα	3.11	(1.19–8.14)	< 0.0001	< 0.0001
IL1β + COX2	1.94	(0.71–5.27)	0.058	IL1β + COX2	4.03	(0.89–18.23)	< 0.0001	< 0.0001
IL1β + TNFα	1.37	(0.71–2.65)	0.212	IL1β + TNFα	3.27	(0.94–11.32)	< 0.0001	< 0.0001
COX2 + TNFα	1.21	(0.79–1.85)	0.293	COX2 + TNFα	2.74	(1.07–7.04)	< 0.0001	< 0.0001
IL-1β + COX-2 + TNFα	6.35	(0.40–101.55)	0.033	IL-1β + COX-2 + TNFα	17.37	(0.54–560.39)	< 0.0001	< 0.0001
Baseline BML	2.43	(1.44–4.08)	< 0.0001	Baseline BML				
Osteophytes MFC	1.42	(0.96–2.08)	0.102	Osteophytes MFC	2.82	(1.12–7.09)	< 0.0001	< 0.0001
Osteophytes MTP	1.01	(0.71–1.45)	0.884	Osteophytes MTP	3.03	(1.20–7.68)	< 0.0001	< 0.0001
Osteophytes LFC	1.80	(1.19–2.73)	0.006	Osteophytes LFC	3.82	(1.485–9.820)	< 0.0001	< 0.0001
Osteophytes LTP	1.25	(0.86–1.81)	0.226	Osteophytes LTP	3.83	(1.452–10.107)	< 0.0001	< 0.0001
Osteophytes MFC + MTP	1.78	(0.76–4.14)	0.212	Osteophytes MFC + MTP	4.72	(1.12–19.87)	< 0.0001	< 0.0001
Osteophytes LFC + LTP	3.35	(1.35–8.30)	0.002	Osteophytes LFC + LTP	9.14	(1.98–42.32)	< 0.0001	< 0.0001
ALL osteophytes	4.20	(0.60–29.65)	0.007	ALL osteophytes	12.10	(0.83–177.02)	< 0.0001	< 0.0001
IL-1β + COX-2 + TNFα + osteophytes MFC + MTP	11.27	(0.27–467.4)	0.038	IL-1β + COX-2 + TNFα + osteophytes MFC + MTP	30.58	(0.37–2532.1)	< 0.0001	< 0.0001
IL-1β + COX-2 + TNFα + osteophytes LFC + LTP	62.06	(1.15–3365.4)	< 0.0001	IL-1β + COX-2 + TNFα + osteophytes LFC + LTP	232.9	(1.85–29,402.2)	< 0.0001	< 0.0001
IL-1β + COX-2 + TNFα + ALL osteophytes	69.48	(0.40–12,199.8)	< 0.0001	IL-1β + COX-2 + TNFα + ALL osteophytes	310.3	(0.67–142,727.2)	< 0.0001	< 0.0001
ALL markers	310.26	(0.67–142,727.2)	< 0.0001					NaN

*PBL* peripheral blood leukocyte, *BML* bone marrow lesion, *JSN* joint space narrowing, *COX-2* cyclooxygenase-2, *IL-1β* interleukin-1 beta, *TNFα* tumor necrosis factor alpha, *95% CI* 95% confidence intervals. All comparisons are versus non-progressors (JSN ≤ 0 mm). Medial (MTP) and lateral (LTP) osteophytes in tibial plateau and medial (MFC) and lateral (LFC) femoral condyle. Total number of progressors (*n* = 66) and non-progressors (*n* = 63) in the combined cohort

**Table 7:** The area under the receiver operating characteristic curves (AUC) comparing baseline *PBL inflammatory gene expression* biomarkers, with and without medial BML, to predict 24-month fast radiographic knee OA progression in the NYU cohort (*N* = 111)

Biomarkers			Biomarkers + baseline medial BML			
Fast progressors (JSN > 0.5 mm) vs. non-progressors (JSN ≤ 0.0 mm)	AUC (95% CI)	Adjusted ***p*** value for model	Fast progressors (JSN > 0.5 mm) vs. non-progressors (JSN ≤ 0.0 mm)	AUC (95% CI)	Adjusted ***p*** value for model	***p*** value for model performance increase
Age	0.41 (0.28–0.53)	0.102	Age	0.69 (0.57–0.80)	0.002	0.001
Sex	0.53 (0.41–0.66)	0.305	Sex	0.62 (0.50–0.74)	0.024	0.021
BMI	0.60 (0.48–0.72)	0.102	BMI	0.67 (0.56–0.79)	0.003	0.102
ASB	0.56 (0.44–0.69)	0.229	ASB	0.65 (0.53–0.77)	0.015	0.075
COX-2	0.68 (0.56–0.80)	0.005	COX-2	0.74 (0.63–0.85)	< 0.0001	0.063
IL-1β	0.57 (0.44–0.69)	0.229	IL-1β	0.65 (0.53–0.77)	0.015	0.073
TNFα	0.55 (0.42–0.67)	0.255	TNFα	0.63 (0.51–0.75)	0.020	0.068
IL1β + COX2	0.58 (0.46–0.71)	0.209	IL1β + COX2	0.65 (0.53–0.77)	0.015	0.109
IL1β + TNFα	0.56 (0.43–0.68)	0.229	IL1β + TNFα	0.64 (0.52–0.76)	0.016	0.073
COX2 + TNFα	0.54 (0.41–0.66)	0.287	COX2 + TNFα	0.63 (0.51–0.75)	0.020	0.066
IL-1β + COX-2 + TNFα	0.67 (0.55–0.79)	0.009	IL-1β + COX-2 + TNFα	0.74 (0.63–0.85)	< 0.0001	0.038
Baseline BML	0.65 (0.53–0.77)	0.020	Baseline BML	0.65 (0.53–0.77)	0.015	1.000
Osteophytes MFC	0.56 (0.44–0.68)	0.229	Osteophytes MFC	0.63 (0.51–0.75)	0.020	0.075
Osteophytes MTP	0.58 (0.45–0.70)	0.220	Osteophytes MTP	0.63 (0.51–0.76)	0.020	0.128
Osteophytes LFC	0.57 (0.44–0.69)	0.229	Osteophytes LFC	0.64 (0.52–0.76)	0.017	0.095
Osteophytes LTP	0.53 (0.41–0.66)	0.309	Osteophytes LTP	0.65 (0.53–0.77)	0.015	0.034
Osteophytes MFC + MTP	0.55 (0.42–0.68)	0.249	Osteophytes MFC + MTP	0.61 (0.49–0.74)	0.035	0.095
Osteophytes LFC + LTP	0.59 (0.46–0.71)	0.209	Osteophytes LFC + LTP	0.65 (0.53–0.77)	0.015	0.072
ALL osteophytes	0.57 (0.44–0.69)	0.229	ALL osteophytes	0.62 (0.49–0.74)	0.035	0.101
IL-1β + COX-2 + TNFα + osteophytes MFC + MTP	0.68 (0.57–0.80)	0.005	IL-1β + COX-2 + TNFα + osteophytes MFC + MTP	0.72 (0.61–0.83)	< 0.0001	0.126
IL-1β + COX-2 + TNFα + osteophytes LFC + LTP	0.70 (0.59–0.82)	0.003	IL-1β + COX-2 + TNFα + osteophytes LFC + LTP	0.75 (0.64–0.85)	< 0.0001	0.074
IL-1β + COX-2 + TNFα + ALL osteophytes	0.70 (0.58–0.81)	0.003	IL-1β + COX-2 + TNFα + ALL osteophytes	0.73 (0.62–0.84)	< 0.0001	0.135
IL-1β + COX-2 + TNFα + BML + ALL osteophytes	0.73 (0.62–0.83)	< 0.0001	IL-1β + COX-2 + TNFα + BML + ALL osteophytes	0.73 (0.62–0.84)	< 0.0001	1.000
Meniscus sum	0.55 (0.43–0.68)	0.244	Meniscus sum	0.63 (0.51–0.75)	0.020	0.084
IL-1β + COX-2 + TNFα + Meniscus sum	0.69 (0.58–0.81)	0.003	IL-1β + COX-2 + TNFα + Meniscus sum	0.73 (0.62–0.83)	< 0.0001	0.152

*ASB* age, sex, and BMI, *PBL* peripheral blood leukocyte, *BML* bone marrow lesion, *JSN* joint space narrowing, *COX-2* cyclooxygenase-2, *IL-1β* interleukin-1 beta, *TNFα* tumor necrosis factor alpha, *95% CI* 95% confidence intervals. Medial (MTP) and lateral (LTP) osteophytes in tibial plateau and medial (MFC) and lateral (LFC) femoral condyle. All comparisons are versus non-progressors (JSN ≤ 0 mm). Total number of progressors (*n* = 44) and non-progressors (*n* = 39)

### Combinatorial biomarkers (medial BML scores and PBL gene expression) enhance prediction of radiographic progression in the combined NYU and OAI cohorts

Having shown that an inflammatory *PBL transcriptome* and BML by MRI individually predicted radiographic progression in our cohorts, we next determined whether any combination of biomarkers had greater predictive value than a single biomarker alone in the combined cohort.

**Fig. 2:**
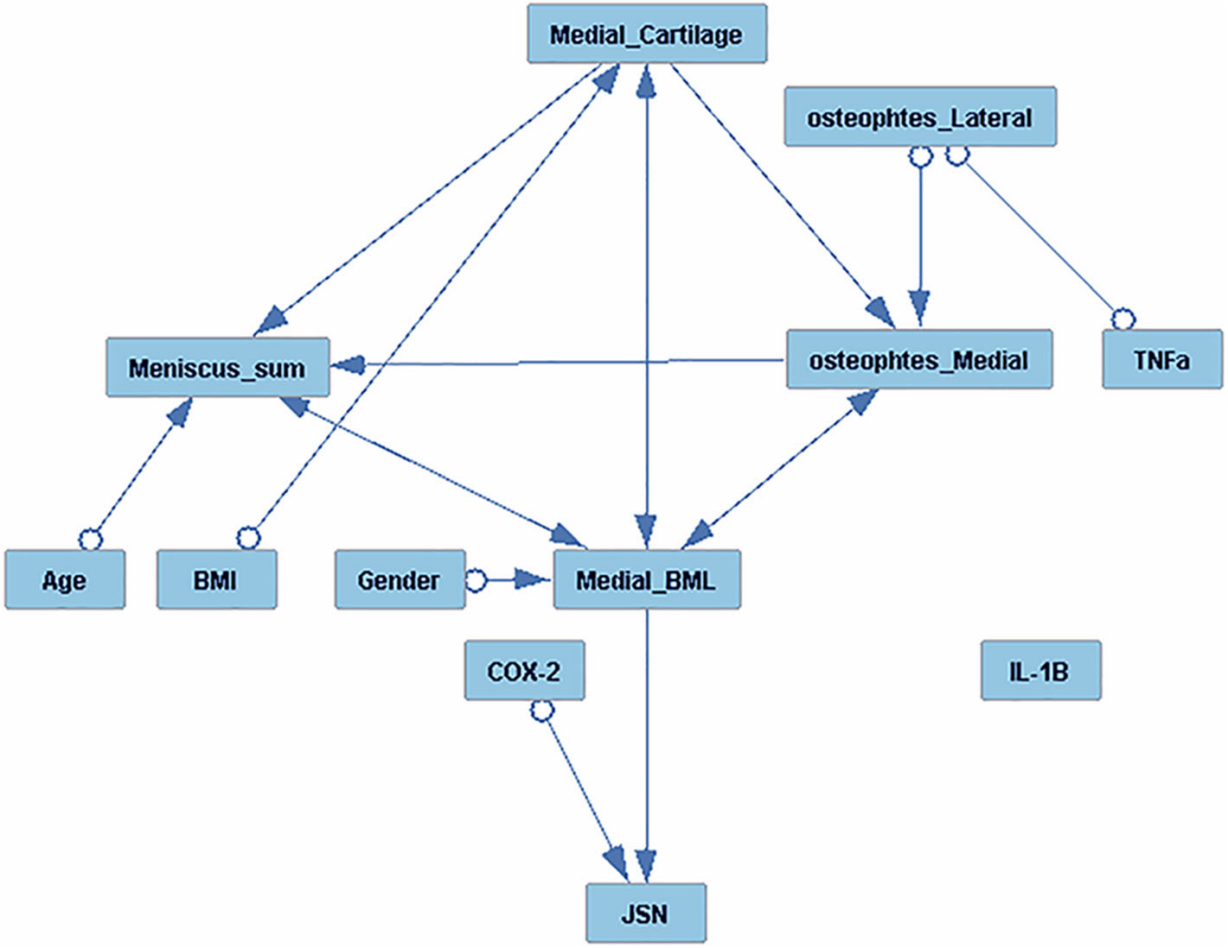
Causal graph analysis of NYU cohort baseline, PBL inflammatory gene expression (*molecular*), X-ray (radiographic), and MRI biomarkers along with age, sex, and BMI on medial JSN. FCI algorithm was used for causal graph analysis of all variables, to determine the interdependence of, inflammatory *PBL gene expression*, radiographic (medial and lateral osteophytes) MRI imaging markers (medial BML, meniscus, and cartilage scores) and covariates (BMI, age, sex) on continuous radiographic JSN over 24 months. Edges with a single arrow denote causality, edges with double arrows denote hidden confounders, and marks (circles) on the edges denote uncertainty of causal orientation. JSN = joint space narrowing; PBL gene expression markers: IL-1β = interleukin-1β; TNFα = tumor necrosis factor α, and COX-2 = inducible cyclooxygenase-2

We first examined the predictive value of the combination of baseline medial BML scores and PBL inflammatory gene expression markers by AUC analyses. As shown in Table 5, the association of age, sex, and BMI alone with fast progressors (JSN ≥ 0.5 mm) was not significant with AUC ranging from 0.46 to 0.54. Moreover, causal analysis (Fig. 2) did not show a direct effect of these variables on joint space narrowing. The association of BML alone with fast progressors (JSN ≥ 0.5 mm) in the combined cohort was AUC = 0.59 (95% CI 0.51–0.67, *p* = 0.035; Table 5). In each instance, the combination of medial BML and individual *PBL inflammatory gene expression* increased the predictive value of either biomarker alone. Specifically, the combination of medial BML and PBL COX-2 expression (AUC 0.65, *p* < 0.0001) yielded the maximal predictive power for fast progressors (Table 5). Additionally, the increased AUC model performance was significant for PBL mRNA transcripts (COX-2, IL1β, and TNFα) in combination with BML (Table 5) in predicting radiographic progression.

In addition to calculating the AUCs, we also determined the odds ratio (OR) for progression for each biomarker, alone and in combination. As shown in Table 6, the OR for baseline PBL COX-2 and IL-1β predicting fast progressors (JSN ≥ 0.5 mm) were 3.56 and 1.90 respectively, and a combination of all three inflammatory PBL gene expression biomarkers (IL-1 β + TNFα + COX-2) had an OR of 6.35. The addition of baseline medial BML to inflammatory PBL IL-1β, COX-2, and TNFα markers increased the OR significantly to 17.37 (*p* < 0.0001).

### Baseline radiographic osteophyte scores and progression of joint space narrowing

Since osteophytes have also been reported to associate with radiographic progression in knee OA [44–47], we added osteophyte assessment to our predictive models in the combined cohort. As shown in Table 6, combination of medial and lateral osteophyte scores increased the OR for progression to 4.2 (*p* = 0.007). The combination of the PBL inflammatory gene expression and osteophyte markers increased the OR to 69.48 (*p* < 0.0001), while the combination of baseline BML, osteophytes, and molecular biomarkers further increased OR to 310.30 (*p* < 0.0001). Similarly, AUC analysis indicated that the predictive value of osteophytes alone significantly increased when combined with PBL gene expression or BML (Table 5).

### Causal graph analysis

To further explore interactions of baseline radiographic (osteophytes) MRI features (medial BML, cartilage, and meniscus), and PBL inflammatory gene expression biomarkers, we performed causal graph analysis to determine the inter-dependent pathways of these factors on continuous JSN over 24 months (Fig. 2). Baseline PBL COX-2 gene expression and baseline medial BML each independently played a causal role or positively influenced JSN. Moreover, age, sex, and BMI did not show a direct effect on joint space narrowing. However, the open circles adjacent to several predictors, including sex, age, BMI, and osteophytes, indicate that there may be hidden confounders, which may influence their relationships with JSN. These new data demonstrate that the PBL inflammatory COX-2 gene expression and medial BML, independently from other MRI or radiographic features, influence radiographic JSN progression.

## Discussion

In the studies here reported, we analyzed two independent cohorts totaling 447 subjects with symptomatic knee OA to determine the prognostic value of baseline PBL inflammatory gene expression, semiquantitative osteophyte score, and MRI features, alone and in combination, as biomarkers of radiographic progression in SKOA. Several observations are noteworthy. First, in an expanded NYU cohort and an independent OAI population, we validated our earlier finding that an inflammatory PBL gene expression is a predictive biomarker of OA radiographic progression, consistent with a state of chronic low-grade inflammation in those at risk for progression [15]. Second, consistent with prior literature, medial BML by MRI and osteophyte score by X-ray were associated with radiographic progression at 2 years. Third, and most importantly, we showed that the combinatorial biomarker of the *molecular*, medial BML, and osteophyte scores markedly increased the predictive value over that of each biomarker alone.

In our analysis of the NYU cohort, we show that baseline medial tibial BMLs were moderately associated with WOMAC and VAS pain, consistent with most prior studies [48, 49]. However, none of the MRI findings were predictive of worsening WOMAC or VAS pain at 2 years. In our study (adjusting for age, sex, and BMI) and in agreement with others [21, 50], we observed a strong inverse correlation of baseline medial BMLs with radiographic JSW and a positive correlation with increased JSN over 24 months (Table 3).

Causal analysis of our data indicates that PBL inflammatory gene expression molecular biomarkers and BML are each independently associated with radiographic progression. Therefore, since these features appear to represent discrete pathogenic processes, it is not surprising that the combination of both molecular and MRI biomarkers significantly increases the prediction of radiographic progression. In addition, we show that the addition of osteophyte scores also enhanced the predictive value of the individual PBL and MRI biomarkers. To our knowledge, this is the first study that has shown the improved prognostic capability for knee OA progression based on a combination of MRI imaging (medial BML), molecular, and radiographic findings.

There have been other recent publications that show the predictive value of molecular *or* imaging biomarkers on progression. A recent FNIH (Foundation of the NIH) study as part of the OA biomarker consortium identified several promising candidates systemic biomarkers such as urine collagen (uCTXII and uCTX1a) as predictors of pain and structural worsening of OA [51]. The final baseline model included uCTXII and sNTXI and had an AUC of 0.586. Similar to the unbiased causal analysis of various biomarkers (Fig. 2) in this study, Loeser and associates have also through an unbiased machine learning approach identified BML, osteophytes, and medial meniscal extrusion as potential biomarkers in identifying radiographic (> 0.7 mm at 48 months) and pain progressors in the OAI cohort [44]. Dunn et al. have studied the peripheral blood methylation status in the radiographic progressors relative to non-progressors in a small cohort of OAI subjects. The epigenetics patterns in peripheral blood mononuclear cells had an AUC of 0.81 in predicting radiographic progressors [52].

What distinguishes our study here is the predictive value of combining molecular biomarkers of inflammation with imaging findings. The combination of biomarkers used together results in predictive values (AUC, odds ratios) for progression that exceed those reported previously for biochemical or imaging alone. In contrast to traditional biochemical markers of cartilage turnover, these data highlight the importance of combining discrete pathogenic events in driving structural progression in OA.

What is the importance of these findings? In addition to shedding light on discrete pathogenic processes, the identification of biomarker predictive of progression is of fundamental importance for the development of new treatment targets through increased efficiencies of trials of disease-modifying agents [51]. There are many obstacles to structure modification studies in OA, including heterogeneity in etiology and the variability in the progression of the disease in clinical trial populations. In ours and prior studies, as many as 25–30% of knee OA patients will not progress over a 2-year study period, and as few as 25–30% will progress at a rate that exceeds 0.5 mm [11–15, 53]. Therefore, in order to adequately power a clinical trial that demonstrates the efficacy of a structure modifying agent, a need exists to identify biomarkers that can identify a population at risk for disease progression. Therefore, prognostic biomarkers have been sought by industry, frustrated by the challenges of drug development in OA. To date, no single prognostic biomarker has been sufficient, and the predictive value of those described have been modest (e.g., OR of 1.2–1.4) [51]. In our studies, we show that the predictive value of an inflammatory PBL gene expression alone is comparable to that of BML by MRI, with OR in the range of 2–4.

One of the limitations of these findings is that none of the biomarkers studied, alone or in combination, predicted symptomatic worsening at 2 years—a time frame chosen to represent a feasible time period for clinical trials. In part, this may be due to the limited period of observation, as compared, for example, to the 48-month period of follow-up in the FNIH study [44]. We note that BML and cartilage readings are semiquantitative, and it is possible that with more advanced scoring systems, precise evaluation of BML and cartilage volume or size would further improve the progression prediction.

Our studies showed variable effects of BMI on progression, observed in the NYU but not the OAI cohort. Demographic differences between the populations included a higher percentage of females in the OAI population, although our sample size were too small to assess the relationship between sex, BMI, and progression. The literature is indeed inconsistent with regard to an independent effect of BMI on radiographic progression of knee OA. While a number publications have shown association of BMI with symptoms and incidence of OA, the literature is mixed regarding a clear association of BMI with OA progression ( [54–57]. An interesting recent article by Wu et al. [58] indicated that the relationship between BMI and progression may also depend on genetics.

. We also note that while the molecular and imaging biomarkers predicted radiographic mJSN at 2 years, neither predicted worsening of pain scores over that interval. This is consistent with reports from the OAI that WOMAC scores do not deteriorate significantly among the subjects followed over several years despite radiographic progression for reasons that remain unclear [44]. The presumption remains that slowing structural progression will prove to be a surrogate for improved function and decreased need for joint replacement at future time points. This is a puzzle yet to be demonstrated, most recently in the FGF-18 trial, which resulted in an improvement in total femorotibial joint cartilage thickness after 2 years but no symptomatic relief was reported [59]; a recent study also reported that cartilage thickness loss was associated with small amount of worsening knee pain [60]. Ideally, however, future treatments will provide both symptomatic relief and attenuate disease progression.

## Conclusions

In summary, we demonstrate that the combination of an inflammatory PBL gene expression (molecular), MRI (BML), and radiographic (osteophyte score) biomarkers significantly enhance the predictive value of any individual biomarker in identifying knee OA patients at risk for radiographic progression. The use of predictive biomarkers to identify an OA population at risk for progression is needed for the future design of disease-modifying OA drug trials and personalized medicine strategies.

## Supplementary information

**Additional file 1.**

## Data Availability

Please contact author for data requests. OAI data are available from OAI.epi-uscf.org

## Abbreviations

OA: Osteoarthritis
SKOA: Symptomatic knee osteoarthritis
KL grade: Kellgren–Lawrence grade
JSW: Joint space width
JSN: Joint space narrowing
BML: Bone marrow lesion
PBL: Peripheral blood leukocyte cells
IL-1β: Interleukin 1 beta
TNFα: Tumor necrosis factor alpha
COX-2: Cyclooxygenase 2
WOMAC: Western Ontario and McMaster Universities Osteoarthritis index
VAS: Visual analog scale
WORMS: Whole-Organ Magnetic Resonance Imaging Score
BMI: Body mass index
AUC: Area under the receiver operating characteristic curves
OR: Odds ratio
