# Supplementary material for: The combination of an inflammatory peripheral blood gene expression and imaging biomarkers enhance prediction of radiographic progression in knee osteoarthritis
Source: Arthritis Res Ther. 2020 Sep 10;22:208. doi: 10.1186/s13075-020-02298-6 (PMC7488029; doi:10.1186/s13075-020-02298-6)
Supplement: Supplementary file 1 — Additional file 1. [file 13075_2020_2298_MOESM1_ESM.docx]

| **NYU (n= 111)** | **Mean (SD)** | **Non-progressors (n=39)** | **Progressors (n=44)** |
| --- | --- | --- | --- |
|  |  | **Mean (SD)** | **Mean (SD)** |
| Age in years | 60.94 (10.22) | 61.41 (8.90) | 60.75 (11.49) |
| sex (% male) | 0.34 (0.66) | 0.31 (0.01) | 0.39 (0.61) |
| BMI (kg/m2) | 26.67 (3.61) | 25.89 (3.77) | 27.36 (3.06) |
| IL-1β | 10.82 (36.06) | 5.23 (9.98) | 18.26 (54.17) |
| COX-2 | 2.46 (4.19) | 1.11 (1.13) | 3.52 (5.75) |
| TNFα | 6.51 (15.66) | 8.19 (24.13) | 5.51 (7.21) |
| Baseline JSW (mm) | 3.65 (1.34) | 3.30 (1.08) | 3.94 (1.58) |
| 24Mon JSW (mm) | 3.13 (1.51) | 3.62 (1.14) | 2.53 (1.77) |
| JSN (mm) | 0.53 (0.89) | 0.32 (0.29) | 1.41 (0.66) |
| osteophytes MFC | 0.50 (0.70) | 0.41 (0.64) | 0.59 (0.69) |
| osteophytes MTP | 0.87 (0.69) | 0.77 (0.63) | 1.00 (0.72) |
| osteophytes LFC | 0.41 (0.64) | 0.31 (0.61) | 0.52 (0.66) |
| osteophytes LTP | 0.51 (0.60) | 0.54 (0.68) | 0.55 (0.55) |
| Baseline medial BML | 1.13 (1.86) | 0.59 (1.14) | 1.78 (2.22) |
|  |  |  |  |
| **OAI (n = 203)** | **Mean (SD)** | **Non-progressors (n=76)** | **Progressors (n=41)** |
|  |  | **Mean (SD)** | **Mean (SD)** |
| Age in years | 62.75 (9.04) | 63.32 (8.91) | 63.59 (8.29) |
| sex (% male) | 0.47 (0.53) | 0.38 (0.01) | 0.44 (0.56) |
| BMI (kg/m2) | 27.33 (3.09) | 27.25 (3.46) | 27.29 (2.66) |
| IL-1β | 1.94 (3.03) | 0.91 (2.03) | 2.34 (2.73) |
| COX-2 | 2.39 (6.49) | 1.36 (1.80) | 4.05 (13.04) |
| TNFα | 2.59 (3.62) | 1.67 (2.51) | 2.90 (2.82) |
| Baseline JSW (mm) | 3.62 (1.30) | 3.86 (1.20) | 3.26 (1.59) |
| 24Mon JSW (mm) | 3.36 (1.49) | 4.05 (1.23) | 2.05 (1.67) |
| JSN (mm) | 0.26 (0.63) | 0.18 (0.16) | 1.20 (0.80) |
| osteophytes MFC | 1.08 (1.17) | 1.12 (1.14) | 1.24 (1.30) |
| osteophytes MTP | 0.93 (1.02) | 1.13 (1.01) | 0.88 (1.10) |
| osteophytes LFC | 0.99 (0.80) | 0.78 (0.64) | 1.37 (0.92) |
| osteophytes LTP | 0.33 (0.38) | 0.38 (0.43) | 0.26 (0.28) |
| Baseline medial BML | 1.90 (2.04) | 1.21 (1.82) | 3.27 (2.31) |
| **NYU+OAI (n= 314)** | **Mean (SD)** | **Non-progressors (n=115)**  **Mean (SD)** | **Progressors (n=85)**  **Mean (SD)** |
| Age in years | 62.11 (9.50) | 62.67 (8.91) | 62.12 (10.12) |
| sex (% male) | 0.42 (0.58) | 0.36 (0.00) | 0.41 (0.59) |
| BMI (kg/m2) | 27.09 (3.29) | 26.79 (3.61) | 27.33 (2.86) |
| IL-1β | 5.29 (22.64) | 2.48 (6.53) | 10.68 (39.85) |
| COX-2 | 2.41 (5.75) | 1.27 (1.59) | 3.77 (9.85) |
| TNFα | 4.02 (10.04) | 3.96 (14.67) | 4.27 (5.69) |
| Baseline JSW (mm) | 3.63 (1.31) | 3.67 (1.19) | 3.61 (1.61) |
| 24Mon JSW (mm) | 3.28 (1.50) | 3.90 (1.21) | 2.30 (1.73) |
| JSN (mm) | 0.36 (0.74) | 0.23 (0.22) | 1.31 (0.74) |
| osteophytes MFC | 0.87 (1.06) | 0.88 (1.05) | 0.91 (1.08) |
| osteophytes MTP | 0.91 (0.92) | 1.01 (0.91) | 0.94 (0.92) |
| osteophytes LFC | 0.78 (0.80) | 0.62 (0.67) | 0.93 (0.90) |
| osteophytes LTP | 0.39 (0.48) | 0.44 (0.53) | 0.41 (0.46) |
| Baseline medial BML | 1.46 (1.97) | 0.83 (1.46) | 2.30 (2.35) |
